# Ethanolic Extract from *Pteris wallichiana* Alleviates DSS-Induced Intestinal Inflammation and Intestinal Barrier Dysfunction by Inhibiting the TLR4/NF-κB Pathway and Regulating Tight Junction Proteins

**DOI:** 10.3390/molecules27103093

**Published:** 2022-05-11

**Authors:** Junhong Tao, Zhihua Huang, Yudan Wang, Yaping Liu, Tianrui Zhao, Yifen Wang, Lei Tian, Guiguang Cheng

**Affiliations:** 1 Faculty of Food Science and Engineering, Kunming University of Science and Technology, Kunming 650500, China; tjh15198526834@163.com (J.T.); sdlcwyd@163.com (Y.W.); liuyaping@kust.edu.cn (Y.L.); food363@163.com (T.Z.); leotian@kust.edu.cn (L.T.); 2 Yunnan Tobacco Company, Yuxi Branch, Yuxi 653100, China; huangzhihua0930@126.com; 3 National and Local Joint Engineering Research Center for Green Preparation Technology of Biobased Materials, Yunnan Minzu University, Kunming 650500, China; 4 Kunming Institute of Zoology, Chinese Academy of Sciences, Kunming 650000, China; wangyifen@mail.kiz.ac.cn

**Keywords:** *Pteris wallichiana*, chemical constituents, ulcerative colitis, anti-inflammatory activity, tight junction proteins

## Abstract

The aim of the research was to determine the protective effect and mechanism of *Pteris wallichiana* J. Agardh extract (PWE) on DSS-induced ulcerative colitis (UC) in mice. In this research, PWE is rich in flavonoids and diterpenoids by UPLC-MS/MS analysis. In LPS-induced RAW264.7 cells, PWE reduced the productions of inflammatory factors (i.e., NO, TNF-α, IL-6, and IL-1β). In DSS-induced UC in mice, PWE improved disease activity index (DAI) score, attenuated oxidative stress by decreasing MPO and MDA activities and activating GSH and SOD levels, and inhibited TNF-α, IL-6, and IL-1β expressions in the colonic tissues. PWE also improved the intestinal barrier by upregulating the expressions of tight junction proteins, including occludin and ZO-1. Moreover, PWE extract alleviated intestinal inflammation by suppressing the TLR4/MyD88/NF-κB signaling pathway. Conclusion: PWE can alleviate DSS-induced UC in mice by increasing the expressions of intestinal tight junction proteins and inhibiting the TLR4/NF-κB inflammatory pathway.

## 1. Introduction

Ulcerative colitis (UC) is an intestinal inflammatory disease that affects the colon and rectum. Its pathological manifestation involves the infiltration of colonic mucosa and submucosal inflammatory cells to deteriorate multiple ulcers [1]. UC had been classified as a refractory disease by the World Health Organization due to its complicated pathology, diagnosis, and treatment [2]. In many countries, the incidence of UC increases every year, and its symptoms can be repeated for several months or years, which reduces the life quality of patients [3]. At present, the main clinical drugs of UC are adrenal cortex hormones, salicylic acids, immunosuppressive agents, and anti-inflammatory drugs. However, these drugs are relatively expensive and have serious side effects (vomiting, headache, diarrhea, hematological disorders, nephrotoxicity, hypokalemia, and risk of infection), and these adverse symptoms seriously affect quality of life [4,5]. On the contrary, traditional Chinese medicine has great advantages in treating UC with significant clinical efficacy [6].

*Pteris wallichiana* J. Agardh is mainly distributed in Yunnan, Sichuan, Guizhou, Tibet, and Guangxi [7]. According to the records of the Medicinal Flora of China, *P. wallichiana* has been used by the Yi nationality as food and in folk medicine, in the treatment of diarrhea, dysentery, and other gastrointestinal diseases [8]. Previous studies reported that *P. wallichiana* is rich in phytochemicals, including carbohydrates, polyphenols, flavonoids, terpenes, etc. [9,10], which are reported to have anti-inflammatory and antioxidant effects [11,12,13]. However, whether *P. wallichiana* consumed as food has therapeutic effects on intestinal diseases has not been reported. Therefore, we investigated the therapeutic effects and mechanisms of *P. wallichiana* in DSS-induced ulcerative colitis (UC) mice. The disease activity index (DAI) scores, colon lengths, and pathological changes were used to evaluate the protective effects of *P. wallichiana* ethanol extract (PWE) in UC mice. The effects of PWE on the expressions of inflammatory factors were detected by an enzyme-linked immunosorbent assay (ELISA). The potential mechanisms of PWE on the inflammatory response and intestinal barrier function were analyzed by using a western blotting assay.

## 2. Results

### 2.1. Chemical Composition of PWE

**Figure 1:**
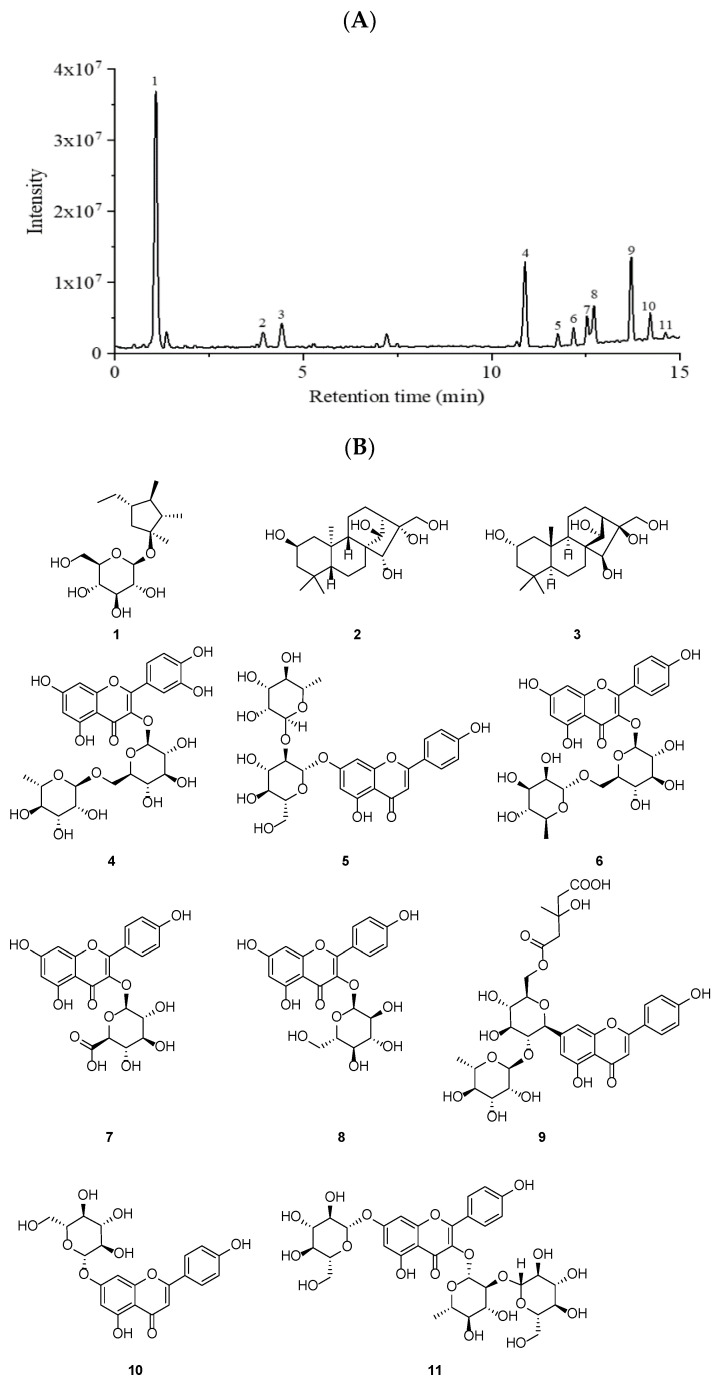
The total ion chromatogram of PWE in the negative mode (**A**). Chemical structure of compounds **1**–**11** (**B**).

The pharmacological effects of each plant were closely related the chemical constituents [14,15]. The TFC, TPC, and TTC in PWE were firstly measured by spectroscopic methods with 89.55 ± 5.23 RE/g extract, 113.65 ± 8.62 mg GAE/g extract, and 10.41 ± 1.44 mg UA/g extract. The chemical compositions of PWE were further identified using an UHPLC-ESI-HRMS/MS analysis. Eleven compounds were identified (Figure 1): sucrose (**1**), 2β,14β,15α,16α,17-pentahydroxy-ent-kaurane (**2**), 2α,14α,15β,16β,17-pentahydroxykaurane (**3**), rutin (**4**), rhoifolin (**5**), kaempferol 3-rutinoside (**6**), kaempferol 3-O-β-D-glucuronide (**7**), kaempferol 3-O-β-D-glucoside (**8**), melitidin (**9**), apigenin 7-O-β-D-glucopyranoside (**10**), and kaempferol-3-sophoroside-7-glucoside (**11**). According to the structural skeleton, the eleven compounds were assigned as eight flavonoids (**4**–**11**), two ent-Kaurane diterpenoids (**2** and **3**), and one sucrose (**1**). The specific information of the identified compounds are summarized in Table 1.

### 2.2. PWE Suppressed Inflammation Response in LPS-Induced RAW264.7 Cells

**Figure 2:**
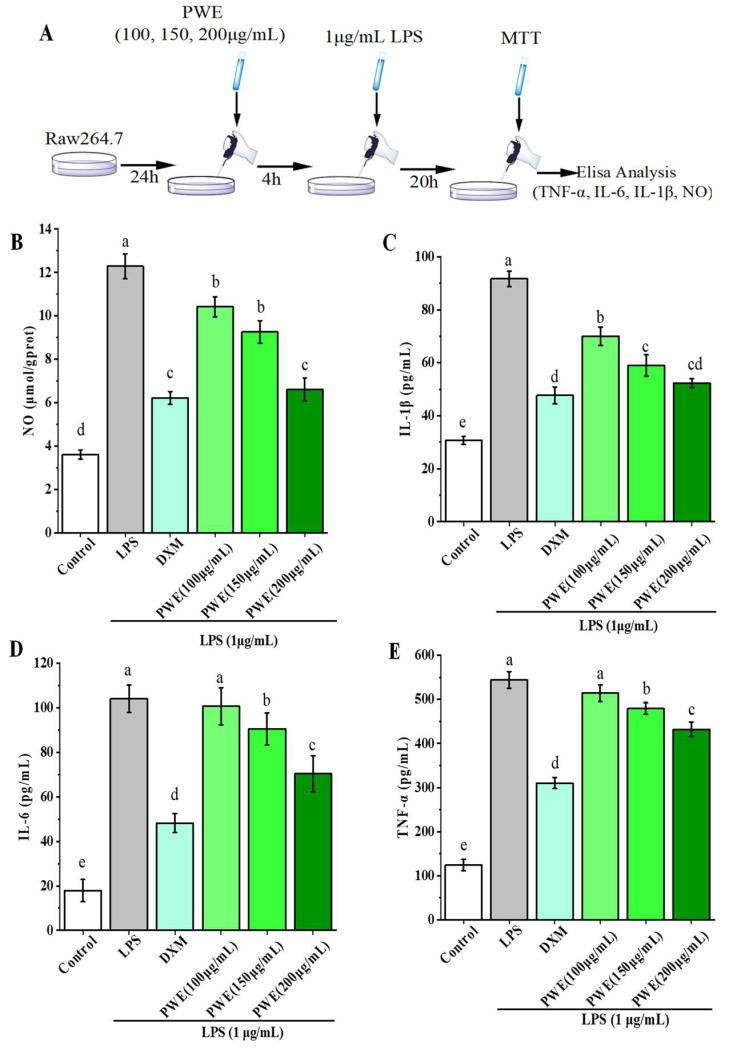
The step diagram of the cell experiment (**A**) and effects of PWE on the production of nitric oxide (NO) (**B**), interleukin-1 beta (IL-1β) (**C**), interleukin-6 (IL-6) (**D**), and tumor necrosis factor-alpha (TNF-α) (**E**) in Raw264.7 cell. Data are presented as means ± SE. Bars with different letters are significantly different (*p* < 0.05). DXM, dexamethasone; LPS, lipopolysaccharides.

An MTT assay showed that PWE had no toxicity to RAW264.7 cells at a dose of 200 µg/mL. Thus, the anti-inflammatory effects of different doses of PWE (100, 150, and 200 µg/mL) on LPS-induced RAW264.7 cells were evaluated by measuring the expressions of TNF-α, IL-1β, IL-6, and NO in the cells. As shown in Figure 2, the contents of TNF-α, IL-1β, IL-6, and NO in LPS-induced RAW264.7 cells were significantly elevated after LPS treatment (*p* < 0.05). Compared with the DSS group, dexamethasone (DXM) decreased NO, IL-1β, IL-6, and TNF-α contents by 49.36%, 48.00%, 53.71%, and 43.01%, respectively (*p* < 0.05). Moreover, the inhibitory effects of 200 µg/mL PWE on NO, IL-1β, IL-6, and TNF-α were 0.94, 0.91, 0.54, and 0.48 times greater than those of DXM, respectively. Therefore, PWE treatment dose-dependently inhibited LPS-induced increases of TNF-α, IL-1β, IL-6, and NO in Raw264.7 cells (Figure 2B–E). In the experiment doses, the above experimental data showed that high-dose PWE had the strongest anti-inflammatory effect.

### 2.3. Acute Toxicity Evaluation of PWE

**Figure 3:**
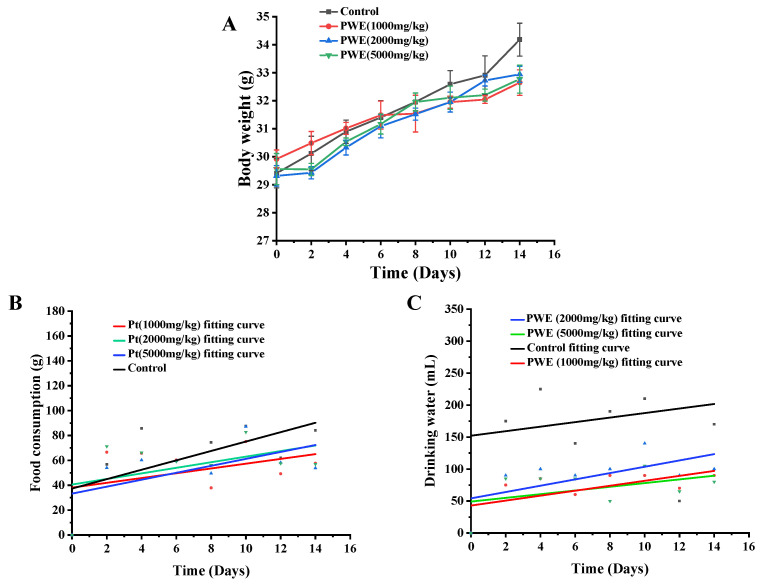
The changes of body weight (**A**), food (**B**), and drinking water (**C**) in acute toxicity evaluation.

Before an animal experiment, the acute toxicity test should be performed to provide the safety dose of the tested sample. In this study, no mortality and abnormal behavior changes (such as fur changes, slow movements, seizures, paralysis) were observed in the PWE treated mice (5000 mg/kg body weight) during the experiment’s 14 days. The organ index of the same organ in each group had no significant difference (Table 2). As shown in Figure 3A, the weight of mice treated with and without PWE increased in the experiment period, and the water and food consumption also increased (Figure 3B,C). Thus, it is safe for mice at a dose of less than 5000 mg/kg PWE.

### 2.4. PWE Administration Attenuated DSS-Induced Ulcerative Colitis in Mice

**Figure 4:**
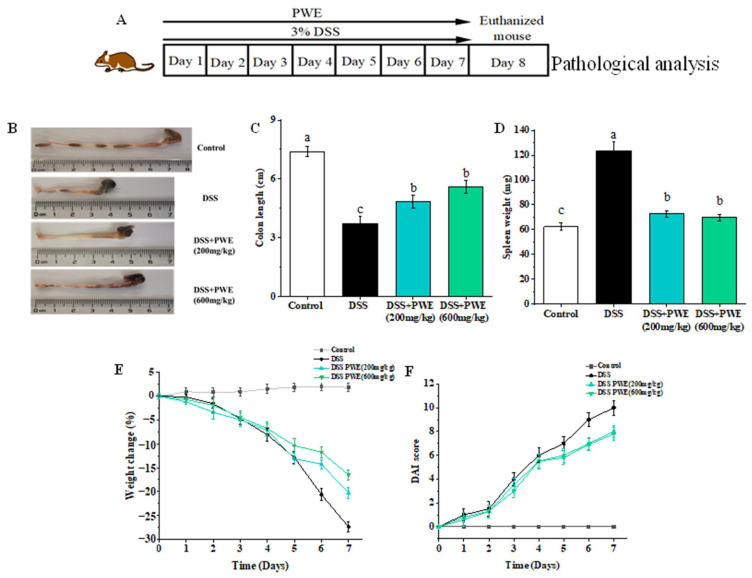
The effects of PWE on improving the symptoms of DSS-induced ulcerative colitis in mice. (**A**) The experiment design. (**B**) Representative images of colon tissues. (**C**) the length of colon tissues in each group (cm). (**D**) Spleen weight in each group. (**E**) Body weight changes throughout this experiment. (**F**) Disease activity index (DAI) score. Data are presented as means ± SE (*n* = 8). Bars with different letters are significantly different (*p* < 0.05).

During the experiment period, the body weight of mice in the control group had no significant difference (Figure 4E). However, the body weight of mice decreased from 23.89 ± 0.47 to 17.35 ± 0.26 g (decreased by 27.37%, *p* < 0.05) the colon length reduced from 7.40 ± 0.26 to 3.73 ± 0.77 cm (shortened by 49.59%, Figure 4A,B), the spleen weight increased from 62.27 ± 3.03 to 123.70 ± 6.78 mg (increased by 49.67%, Figure 4C), and the DAI score increased from 0 to 10 (Figure 4F) (*p* < 0.05) in the DSS group. However, as found in Figure 4C–E, PWE can significantly relieve the decrease of body weight of mice (200 mg/kg: 18.95 ± 0.24 g, 600 mg/kg: 19.75 ± 0.12 g), increase colon length (200 mg/kg: 4.83 ± 0.32 cm, 600 mg/kg: 5.60 ± 0.32 cm), and reduce spleen weight (200 mg/kg: 72.73 ± 2.65 mg, 600 mg/kg: 69.70 ± 0.50 mg). The colon length of the mice in the DSS group reduced by 49.67%, while the PWE low (200 mg/kg) and high (600 mg/kg) dose groups reduced by 34.72% and 24.32% compared with the control group, respectively. The DAI score of the PWE low and high dose groups (200 mg/kg: 0–8, 600 mg/kg: 0–7.5) was significantly (*p* < 0.05) lower than that of the DSS group (Figure 4F). Furthermore, the spleen weight of the mice in the DSS group increased by 49.67%, while the PWE low (200 mg/kg) and high (600 mg/kg) dose groups increased by 14.39% and 10.67% compared with the control group, respectively. These results indicated that the oral administration of PWE could ameliorate the DSS-induced UC symptoms.

### 2.5. PWE Administration Attenuated Histopathological Changes in Colonic Tissues

**Figure 5:**
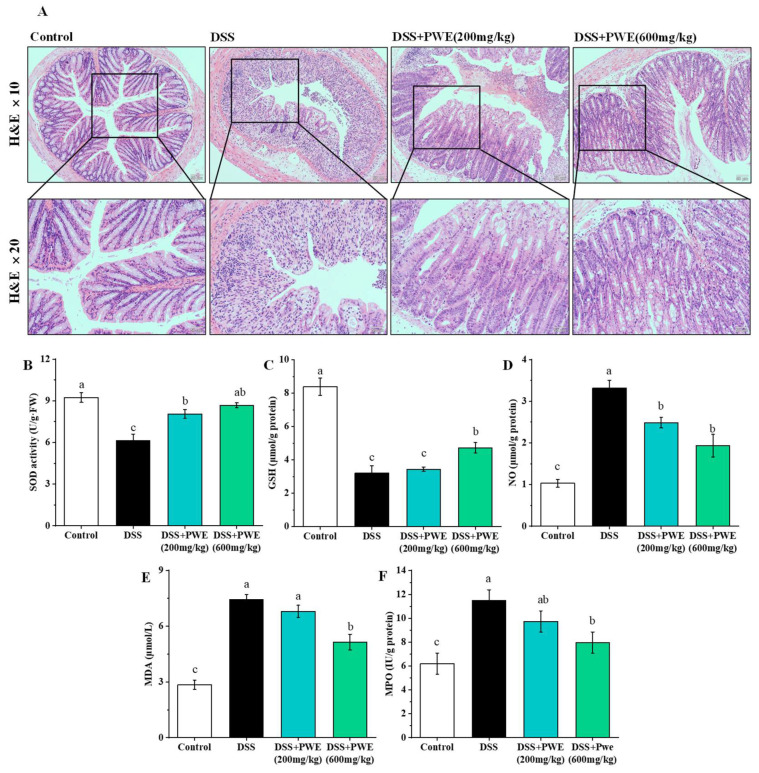
PWE improved intestinal barrier function. H&E-stained images of each group on DSS-induced colon injury in mice (**A**), and effects of PWE on superoxide dismutase (SOD) (**B**), glutathione (GSH) (**C**), myeloperoxidase (MPO) (**F**) activity, malondialdehyde (MDA) (**E**), nitric oxide (NO) (**D**) content in the colon tissue of mice. Data are presented as means ± SE (*n* = 8). Bars with different letters are significantly different (*p* < 0.05).

Histopathological analysis of the colon section is shown in Figure 5A. In the control group, the cells were arranged orderly and there was no epithelial injury and inflammatory cell infiltration. The DSS group significantly damaged the colon, and mainly showed hemorrhage, epithelium mucosal congestion, erosion, a large loss of goblet cells, absence of gland fossae, and a large number of inflammatory cells infiltration. After treatment of PWE, the histopathology of the colon significantly improved. The epithelial cells of the colon were more complete, the crypts were arranged more orderly, and the infiltration of inflammatory cells was less. Moreover, compared with low doses, high doses of PWE can significantly reduce histopathological damage. A histopathological analysis indicated that the oral administration of PWE could protect the damage of colonic tissues.

### 2.6. PWE Ameliorated Oxidative Stress in the Colonic Tissues

The levels of SOD and GSH are shown in Figure 5B,C. The SOD level in the DSS group was dramatically reduced from the 9.23 ± 0.35 to 6.14 ± 0.46 U/g protein (reduced by 33.54%, *p* < 0.05). The SOD levels in the PWE groups at 200 and 600 mg/kg doses were 8.05 ± 0.31 and 8.67 ± 0.18 U per g protein, respectively, which were obviously higher than the DSS group (*p* < 0.05). The GSH level in the DSS-treated group reduced from the 8.39 ± 0.52 to 3.20 ± 0.45 µmol/g protein (reduced by 61.90%, *p* < 0.05). The GSH levels of both the PWE high (600 mg/kg) and low (200 mg/kg) dose groups respectively were 4.73 ± 0.32 µmol/g protein and 3.44 ± 0.14 µmol/g protein, respectively. Moreover, compared to the control group, the level of SOD and GSH in the PWE groups at 200 and 600 mg/kg decreased by 12.90% and 6.07% as well as 58.99% and 43.67%.

The level of MPO, MDA, and NO in the colon is shown in Figure 5D–F, the MPO level in the DSS group increased from 6.19 ± 0.88 to 11.50 ± 0.66 IU/g protein (increased by 46.17%, *p* < 0.05). The MPO levels of both the PWE low and high dose groups were significantly reduced as 9.73 ± 0.41 IU/g protein and 7.96 ± 0.44 IU/g protein, respectively, which were obviously lower than DSS group (*p* < 0.05). Meanwhile, the MDA content of the DSS group increased from 2.85 ± 0.25 to 7.44 ± 0.27 µmol/L of the colon (increased by 61.48%, *p* < 0.05). After PWE treatment, the MDA content of both the PWE low and high dose groups were 6.80 ± 0.32 µmol/L and 5.15 ± 0.42 µmol/L, respectively, which were obviously lower than DSS group (*p* < 0.05) (Figure 4E). The content of NO in DSS group was 3.32 ± 0.08 nmol/gprot, which were obviously higher than control group (1.02 ± 0.05 nmol/gprot) (increased by 69.01%, *p* < 0.05). The NO levels of the PWE low and high dose groups were 2.48 ± 0.28 nmol/gprot and 1.93 ± 0.17 nmol/gprot, respectively. Compared with the control group, the contents of NO, MDA, and MPO in group PWE at 200 and 600 mg/kg increased to 46.79% and 58.63%, 44.55%, and 58.06% as well as 22.24% and 36.38%, respectively. Therefore, PWE can enhance the activities of GSH and SOD in DSS-induced ulcerative colitis mice, and reduce the contents of NO, MDA, and MPO in colon tissue.

### 2.7. PWE Administration Inhibited the Inflammatory Response by TLR-4 Signaling Pathway

**Figure 6:**
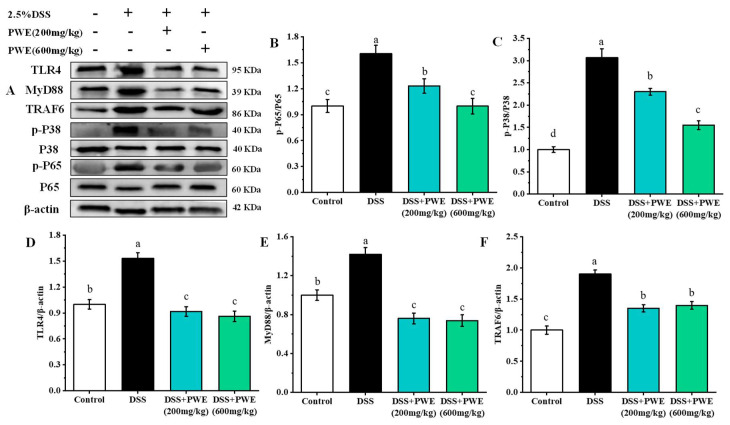
PWE inhibited the activation of the nuclear factor-kappa B (NF-κB) signaling pathway in DSS-induced ulcerative colitis mice. (**A**) Target protein expressions of mice colon. (**B**) Phosphorylated nuclear factor-kappa B p65 protein/nuclear factor-kappa B p65 protein (p-P65/P65), (**C**) phosphorylated p38 mitogen/p38 mitogen (p-P38/P38), (**D**) toll-like receptor 4/β-actin (TLR4/β-actin), (**E**) myeloid differentiation factor 88/β-actin (MyD88/β-actin), (**F**) TNF receptor associated factor 6/β-actin (TRAF/β-actin). Data are expressed as the means ± SE (*n* = 8). Bars with different letters are significantly different (*p* < 0.05).

**Figure 7:**
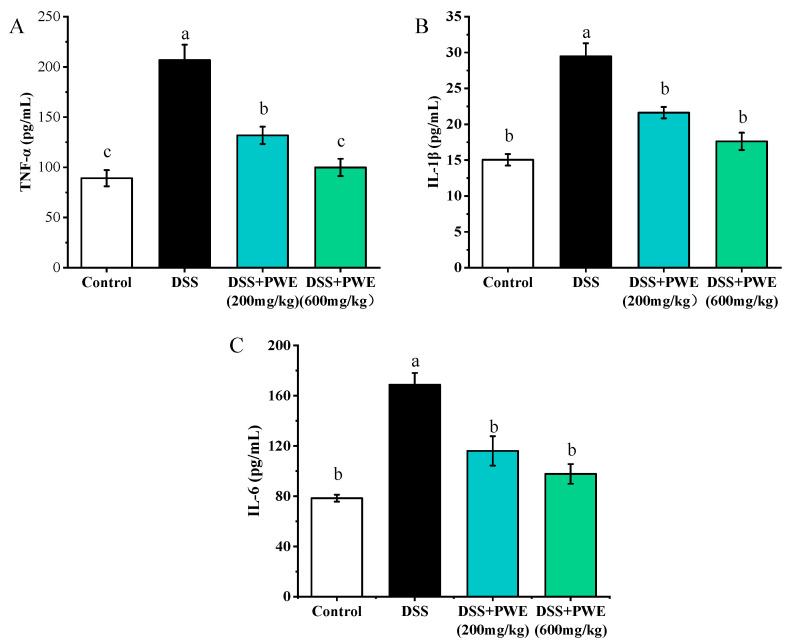
The effects of PWE on the productions of TNF-α (**A**), IL-1β (**B**), IL-6 (**C**) in the different group colon tissues. Data are presented as means ± SE (*n* = 8). Bars with different letters are significantly different (*p* < 0.05). IL-1β, interleukin-1 beta; TNF-α, tumor necrosis factor-alpha; IL-1β, interleukin-1 beta.

The western blotting analysis on the protein expression in the colon tissues is shown in Figure 6. Compared with the control, the expressions of TLR4, MyD88, TRAF6, p-JNK, p-P38, and p-P65 proteins in the DSS group significantly increased (*p* < 0.05). Compared with the DSS group, the expressions of colonic tissue proteins TLR4, MyD88, TRAF6, p-JNK, p-P38, and p-P65 reduced by 37.62%, 49.67%, 0.43, 48.59%, and 26.45% in the PWE (600 mg/kg) group, respectively. Compared with the DSS group, in the DSS + PWE (200 mg/kg), and DSS + PWE (600 mg/kg) groups, the expressions of these proteins significantly reduced in a dose-dependent manner (*p* < 0.05). Additionally, no significant differences were observed in TLR4, MyD88, and TRAF6 expression between the DSS + PWE (200 mg/kg) and DSS + PWE (600 mg/kg) groups (*p* < 0.05). Meanwhile, the levels of inflammatory factors TNF-α, IL-1β, and IL-6 were upregulated in DSS-induced colitis mice (Figure 7A–C). However, PWE treatment could dramatically decrease the expressions of TNF-α, IL-1β, and IL-6 in colon tissues. Compared with the DSS group, the levels of TNF-α, IL-1β, and IL-6 in colonic tissue reduced by 55.66%, 40.24%, 42.06% in the PWE high-dose group, respectively. PWE at 600 mg/kg dose had a stronger inhibitory effect on these inflammatory cytokines than those of low-dose PWE (*p* < 0.05). The above experimental data show that PWE may alleviate colitis by regulating the TLR4/MyD88/TNF-α signaling pathway.

### 2.8. PWE Maintained Intestinal Barrier Function

**Figure 8:**
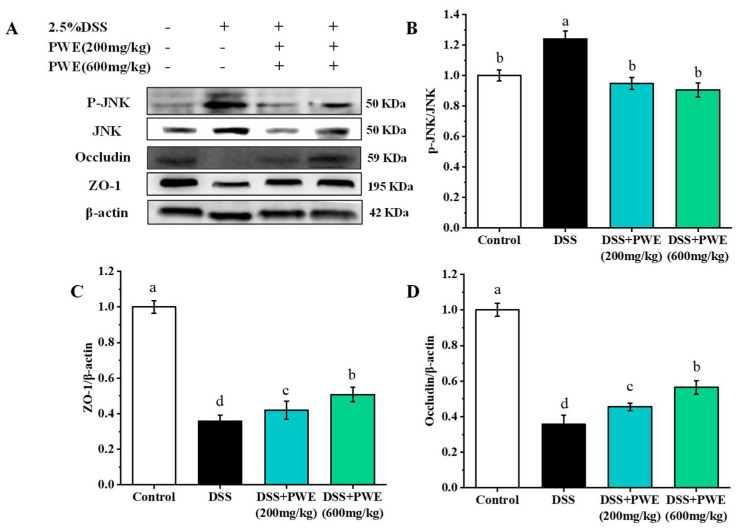
PWE improved the expressions of occludin and ZO-1 by JNK signaling. (**A**) Target protein expressions in mice colon. (**B**) Phosphorylated c-Jun N-terminal kinases/c-Jun N-terminal kinases (p-JNK/JNK), (**C**) zonula occludens-1/β-actin (ZO-1/β-actin), (**D**) occludin/β-actin. Data are expressed as the means ± SE (*n* = 8). Bars with different letters are significantly different (*p* < 0.05).

Tight junctions played a key role in maintaining the cell integrity in the colon tissues. The expressions of ZO-1 and occludin are shown in Figure 8. Compared with the control group, the expressions of occludin and ZO-1 were significantly reduced in the DSS-treated group (*p* < 0.05) (Figure 8A). Compared with the DSS group, the expressions of occludin and ZO-1 protein dose-dependently increased (Figure 8C,D) in the PWE group. Compared with the DSS group, the expressions of ZO-1 and occludin proteins in the colonic tissues increased by 29.53% and 36.70% in the PWE (600 mg/kg) dose, respectively, and (*p* < 0.05). This finding revealed showed that PWE had a protective effect on the alleviation of DSS-induced intestinal barrier dysfunction.

## 3. Discussion

*P. wallichiana* is a traditional ethnic food used in the treatment of gastrointestinal diseases [8]. However, research on the bioactive ingredients and potential therapeutic effects on gastrointestinal diseases is limited. In this study, the chemical component of ethanol extracts of *P. wallichiana* (PWE) was determined. PWE contained polyphenols, flavonoids, and terpenoids at 89.55 ± 5.23 RE/g extract, 113.65 ± 8.62 mg GAE/g extract and 10.41 ± 1.44 mg UA/g extract, respectively. In order to determine the bioactive phytochemicals, a UPLC-MS/MS analysis on PWE was investigated in a negative mode. Eleven compounds were identified, and the main chemical compounds are quercetin derivatives and kaempferol derivatives. This finding is consistent with the reported data in the reference [20]. Previous studies evidenced that the flavonoids from plants had protective effects on inflammatory bowel disease [21,22]. Further, the specific chemical components of PWE are mostly related to the therapeutic effect on UC. Many studies reported that rutin, apigenin 7-O-β-D-glucose, and rhoifolin, had significant antioxidant effects on inhibiting oxidative stress and anti-inflammatory effects via suppressing the expressions of IL-1β, IL-6, and TNF-α [23,24,25]. In addition, quercetin and kaempferol derivatives exhibited inhibitory effects on intestinal inflammation and protective effects on the intestinal barrier [26,27,28]. Thus, the relative higher contents of quercetin and kaempferol derivatives allowed the potential application value in the treatment of UC.

Cristina et al. reported that rutin could play an anti-inflammatory effect in chronic T-lymphocyte-dependent colitis [23]. Chung et al. evidenced that apigenin 7-O-β-D-glucose had antioxidant activity and inhibitory effects on oxidative stress [24]. Fang et al. found that rhoifolin can reduce the expressions of IL-1β, IL-6, and TNF-α in LPS-induced Raw264.7 cells and attenuate acute liver and lung tissue damage in mice by suppressing the inflammatory response. Thus, these flavonoids in PWE may be some of the main active ingredients in the treatment of colitis [25].

In the LPS-indued Raw264.7 inflammation model, PWE could reduce the NO level and the expressions of inflammatory cytokines IL-6, TNF-α, and IL-1β. DSS induction can cause mouse diarrhea, fecal blood, and ultimately lead to mucosal ulcers, colon shortening, and weight loss in mice; the gradual increase in the DAI score is an important indicator for evaluating these symptoms [29]. In DSS-induced colitis mice, PWE can reduce the DAI score, improve DSS-induced weight loss, colonic shortening, and histopathological changes. In histopathological analysis, PWE reduced the inflammatory cell infiltration and mucosal damage in colon tissues. In summary, PWE can relieve colitis in mice and it has a certain therapeutic effect.

Many diseases are closely related to oxidative stress, such as inflammation and cancer. Oxidative stress could be induced by the excessive production of reactive oxygen species and inflammatory factors [30,31]. SOD and GSH are two important enzymes in body tissues and play a vital role in the balance of oxidation and anti-oxidation in the body [14]. In this study, DSS decreased the activities of antioxidant enzymes (GSH, SOD) in vivo, which was consistent with previous studies [32]. However, compared with the DSS group, the levels of SOD and GSH in the PWE group were significantly higher. Therefore, PWE can reduce UC by increasing the activity of SOD and GSH.

MPO was involved in many inflammation regulating processes. MPO-deficient neutrophils were oxidized due to excessive injection into the inflammation site, and formed large amounts of superoxide and oxide, which caused tissue and cell damage in the inflammation sites [33]. Malondialdehyde (MDA) is the final product of lipid peroxidation, so the MDA level could indirectly reflect the cell damage degree [34]. NO is an information molecule that has both a cellular and intercellular messenger and neurotransmitter functions [35]. As another very important free radical in the body, it participated in many pathophysiological processes in the body. In this study, the MPO, MDA, and NO levels increased by DSS treatment. After treatment with PWE, the expressions of MPO, MDA, and NO significantly reduced. In addition, the pathogenesis of UC involved a complex inflammatory response, and eventually the cells could produce excessive amounts of inflammatory cytokines, such as TNF-α, IL-6, and IL-1β. Reducing the expressions of these pro-inflammatory cytokines could improve colitis in mice. In this experiment, PWE treatment significantly inhibited the increase of TNF-α, IL-6, and IL-1β in inflammatory cytokines induced by DSS to a certain extent.

Previous studies have found that the TLR4 activation and the NF-κB translocating into the nucleus were two very important mechanisms for regulating inflammatory diseases [36,37]. The TLR4 activation can produce a large amount of myeloid differentiation primary response protein 88 (MyD88), which further activates the ubiquitin ligase TRAF6. TRAF6 can activate the NF-κB and MAPK signaling pathways [38]. Therefore, NF-κB is an important regulatory protein for cellular inflammation-related genes transcription [39]. Regulation of the TLR4/MyD88/NF-κB signaling pathway may be an important mechanism for the treatment of colitis inflammation in mice [40]. Western blotting analysis showed that DSS-induced overexpression of TLR4, MyD88, and TRAF6 in the colon tissue were significantly inhibited after PWE treatment (Figure 6). Correspondingly, the p65 phosphorylation was also significantly inhibited. Finally, NF-κB promoted the inflammatory factor expression and secretion after entering in the nucleus [41]. According to the downregulation effect on the inflammatory factors TNF-α, IL-1β, and IL-6 in colon tissue, it was proved that PWE could inhibit the TLR4/MyD88/NF-κB pathway activation, which thereby inhibited the expressions of the inflammatory factors.

Ulcerative colitis is mainly related to the colonic mucosa and submucosa lesions. The intestinal epithelial barrier plays an important role in intestinal diseases [42]. Tight junction (TJ) protein is an important functional protein in the intestinal epithelial barrier, which could prevent pathogenic antigens from entering the lamina propria [43]. Occludin is one of the transmembrane tight junction proteins responsible for regulating the intestinal permeability [44]. ZO-1 was an important member of the peripheral membrane protein in the TJ protein. It was mainly involved in regulating the transport of cellular material and maintaining the integrity of the TJ complex [45]. Tight junction proteins (ZO-1, occludin, etc.) may be indicators used to evaluate the degree of intestinal inflammation and predicting mucosal healing in patients with ulcerative colitis.

The JNK signaling pathway is participated in many physiological and pathological functions, such as cell cycle, apoptosis, and cell stress. JNK phosphorylation could cause the decomposition of the tight junction complex for destroying the intestinal barrier function and increasing the intestinal permeability [46]. In the current work, expression of protein analysis showed that compared with the DSS group, PWE administration increased the expressions of ZO-1 and the occludin protein in colon tissues of DSS-treated colitis mice in a dose-dependent manner. PWE treatment also inhibited the JNK phosphorylation. This finding showed that PWE may increase the expressions of TJ proteins by inhibiting the activation of JNK, thereby maintaining the integrity of the intestinal mucosal barrier.

This study revealed the molecular mechanism of anti-inflammatory effects of active natural ingredients in *P. wallichiana* provided theoretical guidance for the application of *P. wallichiana* in colitis, provided scientific basis for the prevention, treatment and drug development of inflammatory diseases, such as UC, and provided a theoretical basis for the development of *P. wallichiana* in functional food. *P. wallichiana*, the edible and medicine plant in the Yi minority, has a series of pharmacological activities for human health. The present study just investigated the therapeutic effect for 80% ethanol aqueous extract from *P. wallichiana* on ulcerative colitis. However, the bioactive agents for *P. wallichiana* in the protection of ulcerative colitis were unknown. Therefore, exploration of the specific bioactive agents in *P. wallichiana* is needed for further investigation.

## 4. Materials and Methods

### 4.1. Plant Materials and Extraction

*P. wallichiana* was collected from Ninglang county of Lijiang (GPS coordinates: 27°11′ N/100°41′ E) in Yunnan Province, and was identified by Professor Cao Jianxin of Kunming University of Science and Technology. A specimen (no. Cheng20191021-08) was stored at the Faculty of Food Science and Engineering, Kunming University of Science and Technology (http://www.theplantlist.org/tpl1.1/record/tro-26600312, accessed on 21 October 2019). According to previous studies, the aerial parts of *P. wallichiana* were dried by the shade-drying method and then powdered by using a pulverizer [47]. Then, the sample was extracted with 80% ethanol aqueous solution for three times (30 min each time) by an ultrasonic assisted extraction method at room temperature [48,49]. After that, the extraction solution was collected and condensed by a rotary evaporator under a vacuum condition for removing the ethanol. The residue aqueous solution was degreased by using petroleum ether at a ratio of 1:1 (*v*/*v*) for three times. Finally, the water phase was concentrated by using a rotary evaporator, further dried by using a lyophilizer (Alpha 1–2 LD plus, Christ, Osterode, Germany). Then the extract was stored in a −20 °C refrigerator.

### 4.2. Determination of the Contents of Total Phenolics, Flavonoids, and Terpenoids

The total phenolic content (TPC), total flavonoid content (TFC), and total terpenoid content (TTC) were determined according to a reported method previously described Folin-Ciocalteu method [50,51], colorimetric method [52], and vanillin-glacial acetic acid-perchloric acid method [53], respectively. The TPC was expressed as mg gallic acid (GE)/g dry extract. The TFC was expressed as mg rutin (RE)/g dry extract. The TTC was expressed as mg ursolic acid (UC)/g dry extract.

### 4.3. Identification of Chemical Constituents in PWE by UHPLC-ESI-HRMS/MS

The chemical composition of PWE was analyzed on a Thermo Scientific Q Exactive Orbitrap LC/MS System. The water phase after being dried by using a lyophilizer was dissolved in methanol (100 ug/mL) and analyzed on a Shimadzu Shim-pack GIST C18 column (2.1 mm × 100 mm, 3 μm, Shimadzu, Kyoto, Japan). Ultrapure water (solvent A) and acetonitrile (solvent B) were used as mobile phases, and the mobile phase was added 0.1% formic acid (*v*/*v*). The gradient was as follows: 0–3 min, 10% B; 3–20 min, 10–40% B; 20–25 min, 40–60% B; 25.01–30 min, 100% B. The flow rate of mobile phases was 0.2 mL/min, the sample volume was 2.0 µL, and the temperature of the column oven was maintained at 35 °C. Data were collected in a negative ion mode. The mass range of the scan was *m*/*z* 50 to 1000. Other parameters for mass spectrometry were as follows: auxiliary gas flow, 8.0 L/min; sheath gas flow rate, 32.0 L/min; sweep gas, 4.0 L/min; spray voltage, 3.3 kV; S-lens RF level, 50%; resolution, 70,000; heater temperature, 350 °C; and capillary temperature, 320 °C. The total ion chromatogram, mass data, and MS/MS fragment ion data were analyzed by Xcalibur software. The identification of each compound in the PWE was compared with the reported data in the literature or the Metabolomics Innovation Center database.

### 4.4. Cell Culture and Viability Assay

RAW264.7 cells were purchased from Kunming Institute of Zoology, Kunming, China. RAW264.7 cells were cultured in a culture medium containing 10% fetal bovine serum (FBS) and 50 U/mL penicillin and streptomycin in Dulbecco’s Modified Eagle Medium (DMEM). The incubator culture conditions included a concentration of 5% CO_2_ and 37 °C. RAW264.7 cells in the logarithmic growth phase were diluted to a density of 2 × 105 cells/well in 96-well cell culture plates for 24 h. Then different concentrations of PWE (100, 150, and 200 µg/mL) were added to the plates for 20 h of incubation. After that, 20 µL of MTT (5 mg/mL) was added to each well. After 4 h of incubation, the cell culture medium was removed. After adding dimethyl sulfoxide (DMSO) (150 µL/well) solution, the cell spectrophotometric value was detected at 570 nm using a microplate reader.

### 4.5. Cytokine Assays

After the cytotoxic evaluation, RAW264.7 cells in logarithmic growth phase were cultured in 6-well plates (2 mL/well) at a density of 2 × 10^5^ cells/well for 24 h. Then different concentrations of PWE (100, 150, 200 µg/mL) were respectively added to RAW264.7 cells in each group. After 2 h of incubation, the cells were stimulated with 2 mL lipopolysaccharide (LPS) (1 µg/mL) for 20 h. The supernatant was centrifuged and collected. The colon tissues were broken and homogenized with 10% normal saline solution; the supernatant was obtained after centrifugation at 10000 r/min for 10 min. The levels of tumor necrosis factor alpha (TNF-α), nitric oxide (NO), interleukin-1β (IL-1β), interleukin-6 (IL-6) in RAW264.7 cells supernatant, and colon tissue supernatant were measured by enzyme linked immunosorbent assay (ELISA) kits (Nanjing Jiancheng Bioengineering Institute, Nanjing, China) following the manufacturer’s instructions.

### 4.6. Animals

A total of 40 C57BL/6 male mice (21–23 g) and 40 Kunming female mice (21–23 g) were provided by the Center of Laboratory Animal Science Research of Kunming Medical University (Kunming, China) and raised under specific pathogen-free (SPF) conditions: temperature (25 ± 1 °C), humidity (55 ± 5%), 12 h light/dark cycles, sufficient distilled water and standard chow diet (AIN-93G). All mice were adapted for one week under the conditions of sufficient water and food. All experimental procedures were performed in accordance with the National Institute of Health Guide for the Care and Use of Laboratory Animals, and were approved by the Ethical Committee for Animal Experimentation of Kunming University of Science and Technology (SLWH(Dian)K2021-0007).

### 4.7. Acute Toxicity Assays

The acute toxicity assay was performed as our previously reported method [54]. The female mice were randomly divided into four groups (*n* = 10): control group, with three different doses of PWE with 1000, 2000, and 5000 mg/kg, respectively. Mortality and behavior changes of the mice (such as fur changes, slow movements, seizures, paralysis, and water and food consumption) were recorded every day. After 14 days of experiments, the mice were euthanized, the key organ tissues of the mice were collected, and the organ index was calculated. The lethal dose (LD50) was computed by the early-stage methods of this laboratory [55].

### 4.8. Animal Experiment Design of DSS-Induced Ulcerative Colitis

The male C57BL/6 mice were randomly divided into four groups (*n* = 10): control group, DSS group, DSS + PWE (200 mg/kg), and DSS + PWE (600 mg/kg). The mice, except the control group, received 3.0% dextran sodium sulfate (DSS) solution for 7 consecutive days, through oral administration. The mice in the control group were given the same volume of distilled water. The dose of PWE was selected based on preliminary experiments and acute toxicity experiments. In PWE-treated groups, the mice were orally administrated with doses of 200 mg/kg and PWE 600 mg/kg per day. The weight of each mouse was recorded and the colitis disease activity index (DAI) was assessed every day [56]. After given DSS for 7 days, the animals were euthanized after fasting for 12 h. The length of the colon tissue was measured, while the spleen was collected and then weighed for calculating the organic index. The remaining organs were stored in a refrigerator at −80 °C for subsequent analysis.

### 4.9. Hematoxylin and Eosin (H&E) Staining

A portion of the distal colon tissue was fixed in 4% buffered paraformaldehyde overnight, embedded in paraffin, and cut into four µm-thick sections. After deparaffinization, the sections were stained with hematoxylin and eosin (H&E). Colon tissue histopathology was assessed according to a previous described method [57].

### 4.10. Determination of GSH, SOD, MPO, MDA, and NO Content in Colon Tissues

The colon tissue was rinsed with phosphate buffer solution, homogenized in tissue lysate, and centrifuged at 6700 g for 10 min at 4 °C. The supernatant was collected for further analysis. The variations of glutathione (GSH) activity, superoxide dismutase (SOD) activity, myeloperoxidase (MPO) activity, malondialdehyde (MDA) level, and the nitric oxide (NO) level in the colon tissue were measured, according to the instructions of the purchased enzyme-linked immunosorbent assay (ELISA) kit (Nanjing Jiancheng Bioengineering Institute, Nanjing, China).

### 4.11. Western Blotting Analysis

Western blot analysis is based on previous research [58]. The colon tissues were mixed with lysis buffer, and then crushed and homogenized in a crusher, centrifuged at 6700 *g*/10 min, and absorbed supernatant. Antibodies of zonula occludens-1 (ZO-1), occludin, toll-like receptor 4 (TLR4), myeloid differentiation factor 88 (MyD88), TNF receptor associated factor 6 (TRAF6), c-Jun N-terminal kinases (JNK), p38 mitogen-activated protein kinase (P38), nuclear factor-kappa B p65 protein (NF-κB), and β-actin were diluted according to the manufacturer’s instructions (Servicebio Technology Co., Ltd., Wuhan, China). The proteins in colon tissue lysates were separated by an SDS-polyacrylamide gel and electro-transferred onto polyvinylidene difluoride (PVDF) membranes. After that, membranes were blocked by 5% skim milk for 1 h, and then incubated with diluted primary antibody for two hours at room temperature. The polyvinylidene difluoride (PVDF) membranes were incubated with a horseradish peroxidase (HRP)-conjugated secondary antibody for 1 h at 25 °C. After adding the chemiluminescence substrate, the bands were displayed on the chemiluminescence imaging system.

### 4.12. Statistics Analyses

The experimental data are expressed as mean ± standard error (SE). In the process of data processing, Origin 2019 b and SPSS 19.0 software were used for statistical test or one-way ANOVA. Experimental data were repeated no less than three times. *p <* 0.05 indicates statistically significant.

## 5. Conclusions

This experiment studied the chemical components of PWE and its protective effect on DSS-induced colitis; 2β,14β,15α,16α,17-pentahydroxy-ent-kaurane, 2α,14α,15β,16β,17-pentahydroxykaurane, rhoifolin, rutin, kaempferol 3-rutinoside, kaempferol 3-O-β-D-glucuronide, kaempferol 3-O-β-D-glucoside, melitidin, apigenin 7-O-β-D-glucopyranoside, and kaempferol-3-sophoroside-7-glucoside were the main chemical constituents. In LPS-induced Raw264.7 inflammation and DSS-induced mouse colon UC, PWE pretreatment can significantly reduce the expressions of inflammatory cytokines in vitro and in vivo. In DSS-induced UC mice, PWE can improve the abnormal pathological tissue damage by inhibiting oxidative stress and inflammation-related signaling pathways, and improve the intestinal barrier function. The results show that *Pteris wallichiana* can be developed into functional foods or health products for the prophylaxis or control of intestinal diseases, such as colitis.

## Figures and Tables

**Table 1:** Qualitative analysis of the chemical constituents in PWE by UHPLC-ESI-HRMS/MS.

Peak No.	Rt *^#^* (Min)	Compounds	Molecular Formula	[M-H]^-^ *m*/*z*	MS/MS Fragment Ions	Ref.
1	1.08	sucrose	C_12_H_22_O_11_	341.1096	59.0126, 71.0126, 101.0232, 323.4808	HMDB *
2	3.94	2β,14β,15α,16α,17-pentahydroxy-ent-kaurane	C_20_H_34_O_5_	353.0886	85.0283, 135.0443, 173.0449, 179.0345	[16]
3	4.43	2α,14α,15β,16β,17-pentahydroxykaurane	C_20_H_34_O_5_	353.0887	93.0334, 135.0443, 173.0449, 191.0557	[16]
4	10.89	Rutin	C_27_H_30_O_16_	609.1476	151.0026, 301.0353, 343.0476, 609.1475	standard
5	11.75	Rhoifolin	C_27_H_30_O_14_	577.1578	102.9558, 285.0410, 431.0981,577.1572	HMDB *
6	12.17	Kaempferol 3-rutinoside	C_27_H_30_O_15_	593.1527	151.0027, 285.0408, 327.0510, 593.1530	standard
7	12.52	Kaempferol 3-O-β-D-glucuronide	C_21_H_18_O_12_	461.0737	85.0283, 113.0234, 285.0410, 389.3503	[17]
8	12.71	Kaempferol 3-O-β-D-glucoside	C_21_H_20_O_11_	447.0944	151.0028, 284.0329, 300.0280, 447.0925	HMDB *
9	13.71	Melitidin	C_33_H_40_O_17_	7235045	73.7354, 347.8025, 451.3281, 677.4988	[17]
10	14.21	Apigenin 7-O-β-D-glucopyranoside	C_21_H_20_O_10_	431.0992	227.0351, 255.0302, 284.0331, 431.0987	[18]
11	14.62	Kaempferol-3-sophoroside-7-glucoside	C_33_H_40_O_21_	771.1798	151.0038, 301.0359, 609.1474, 771.1782	[19]

*^#^*: Rt: retention time; *: HMDB: Human Metabolome Database.

**Table 2:** Organ index of mice in acute toxicity test.

Organ	Control (g/100 g)	PWE (1000 mg/kg) (g/100 g)	PWE (2000 mg/kg) (g/100 g)	PWE (5000 mg/kg) (g/100 g)
Heart	0.54 ± 0.04	0.53 ± 0.03	0.54 ± 0.06	0.55 ± 0.02
Liver	4.48 ± 0.45	4.52 ± 0.56	4.47 ± 0.43	4.50 ± 0.38
Spleen	0.35 ± 0.02	0.36 ± 0.05	0.34 ± 0.03	0.36 ± 0.07
Lung	0.69 ± 0.06	0.70 ± 0.03	0.71 ± 0.06	0.68 ± 0.12
Kidney	1.08 ± 0.09	1.12 ± 0.02	1.10 ± 0.01	1.12 ± 0.04
Thymus	0.46 ± 0.04	0.48 ± 0.05	0.45 ± 0.05	0.47 ± 0.03

## Data Availability

The data that support the findings of this study are available from the corresponding author upon reasonable request.
